# Antitumor activity of vorinostat-incorporated nanoparticles against human cholangiocarcinoma cells

**DOI:** 10.1186/s12951-015-0122-4

**Published:** 2015-09-26

**Authors:** Tae Won Kwak, Do Hyung Kim, Young-Il Jeong, Dae Hwan Kang

**Affiliations:** Biomedical Research Institute, Pusan National University Hospital, 179 Gudeok-ro, Seo-gu, Busan, 602-739 Republic of Korea; School of Medicine, Pusan National University, Yangsan, Gyeongnam 626-770 Republic of Korea; Department of Internal Medicine, Pusan National University Yangsan Hospital, Yangsan, Gyeongnam 626-770 Republic of Korea

**Keywords:** Vorinostat, Cholangiocarcinoma, Nanoparticles, Poly(dl-lactide-co-glycolide), Block copolymer, Cancer chemotherapy, Drug targeting

## Abstract

**Background:**

The aim of this study is to evaluate the anticancer activity of vorinostat-incorporated nanoparticles (vorinostat-NPs) against HuCC-T1 human cholangiocarcinoma cells. Vorinostat-NPs were fabricated by a nanoprecipitation method using poly(dl-lactide-co-glycolide)/poly(ethylene glycol) copolymer.

**Results:**

Vorinostat-NPs exhibited spherical shapes with sizes <100 nm. Vorinostat-NPs have anticancer activity similar to that of vorinostat in vitro. Vorinostat-NPs as well as vorinostat itself increased acetylation of histone-H3. Furthermore, vorinostat-NPs have similar effectiveness in the suppression or expression of histone deacetylase, mutant type p53, p21, and PARP/cleaved caspase-3. However, vorinostat-NPs showed improved antitumor activity against HuCC-T1 cancer cell-bearing mice compared to vorinostat, whereas empty nanoparticles had no effect on tumor growth. Furthermore, vorinostat-NPs increased the expression of acetylated histone H3 in tumor tissue and suppressed histone deacetylase (HDAC) expression in vivo. The improved antitumor activity of vorinostat-NPs can be explained by molecular imaging studies using near-infrared (NIR) dye-incorporated nanoparticles, i.e. NIR-dye-incorporated nanoparticles were intensively accumulated in the tumor region rather than normal one.

**Conclusions:**

Our results demonstrate that vorinostat and vorinostat-NPs exert anticancer activity against HuCC-T1 cholangiocarcinoma cells by specific inhibition of HDAC expression. Thus, we suggest that vorinostat-NPs are a promising candidate for anticancer chemotherapy in cholangiocarcinoma.

**Graphical abstract:**
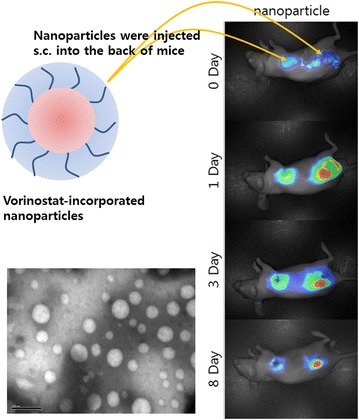
Local delivery strategy of vorinostat-NPs against cholangiocarcinomas.

## Background

Vorinostat (suberoylanilide hydroxamic acid, SAHA) is known to one of histone deacetylase inhibitor (HDACi) [1]. In acetylation process, acetyl group in one molecule is transferred to another and deacetylation reaction is to remove acetyl group from a molecule. HDAC has an important role in the transcriptional regulation through stabilization of DNA-histone interaction and deacetylation process is known to have relationship with carcinogenesis [1]. HDACis such as vorinostat act as a chelator for zinc ions in the active site of histone deacetylases (HDACs) and vorinostat is regarded as a promising cancer chemotherapeutic drug [1]. Vorinostat has been approved by the FDA for treatment of cutaneous T cell lymphoma [1, 2]. Accumulation of acetylated histones and acetylated proteins has correlation with *p21*^*WAF1*^ gene expression, apoptotic signals such as mutant-type *p53* and active-type caspase expression, cell differentiation and cell death [1–5]. In recent clinical trials, the safety and anticancer efficacy vorinostat has been evaluated against gastrointestinal (GI) cancer patient [3]. In the results of these trials, the report suggested that vorinostat can be used as an effective anticancer agent for GI cancer [3]. Vorinostat induced both apoptosis and autophagy in gastric cancer cell lines and has shown clinical benefits for gastric cancer patients [6, 7]. The anticancer activity of vorinostat has also investigated against colon cancer, glioma, lung cancer, breast cancer and hepatocellular carcinoma in preclinical or clinical trials, both as a single treatment or combination with other types of anticancer drugs [5–8]. We previously reported that vorinostat exhibits anticancer efficacy against HuCC-T1 human cholangiocarcinoma (CCA) cells [9]. In this report, we show that vorinostat is involved in growth inhibition, apoptosis of HuCC-T1 cells in vitro and anti-tumor activity of HuCC-T1 cell-bearing xenograft model in vivo.

CCA is a malignant tumor that occurs in the epithelium of the biliary tract [10]. Although the rate of incidence of CCA has increased worldwide, the reason for its increase remains unclear [11, 12]. Current treatment options for CCA include surgical resection, radiotherapy, chemotherapy, stent displacement and immunotherapy [13–15]. Although surgical resection is believed to be a curative treatment option for CCA, patients with CCA are frequently diagnosed at an unresectable stage [16]. Chemotherapeutic approaches for CCA are considered to increase patient survival and quality of life [12]. Various chemotherapeutic agents such as gemcitabine, cisplatin, oxaliplatin, capecitabine and 5-fluorouracil have been tested as single agents or in combination in clinical trials for CCA [17, 18]. Even though the combination of some anticancer agents have been reported to have therapeutic advantages, systemic chemotherapy using conventional anticancer agents is still ineffective and shows an insignificant increase in survival period. In fact, current standard chemotherapeutic treatment for CCA patients is normally gemcitabine plus cisplatin [18, 19]. Even though combination of these chemotherapeutic agents delayed onset of progression, most cases still succumbed to CCA and has no significant advances in survivability [20]. Because most of chemotherapeutic agents showed minimal survival gain and chemotherapeutic agents have difficulties in delivery to CCA, targeted therapy for CCA patients has been proposed [21]. Novel treatment options for a chemotherapeutic approach for CCA are required to improve patient survivability.

Nanomedicine such as nanoparticles, liposomes and polymeric micelles have advantages in targeting malignant solid tumor because they have small sizes of <1000 nm and unique structures that can amplify the anticancer activity of conventional drugs [22–27]. In recent decades, nanomedicine-based drug delivery systems have also been investigated to target CCA cells for diagnosis and chemotherapeutic treatment [22–27]. Magnetic nanoparticles were reported to be a useful device for the diagnosis of intrahepatic CCA [22, 23]. Magnetic drug nanoparticles enveloping chemotherapeutic drugs were reported to be an effective treatment for the inhibition of CCA cell proliferation in a tumor xenograft model of nude mice [24]. Totawa et al. reported that hybrid liposomes were specifically accumulated in human CCA cells and induced cell cycle arrest [25]. In our previous study, chitosan nanoparticles incorporating all-trans retinoic acid were demonstrated to be effective in inhibiting the invasion, migration and proliferation of human CCA cells [26]. Stimuli-sensitive nanoparticles can also be considered to target CCA cells [27].

In this study, we prepared vorinostat-NPs using biodegradable polymers to assess their anticancer effects on HuCC-T1 cells in vitro and in vivo. The efficacy of vorinostat and vorinostat-NPs in HuCC-T1 cells was studied using western blotting, immunohistochemistry and a HuCC-T1 xenograft model in nude mice.

## Results

### Characterization of vorinostat-incorporated nanoparticles

**Fig. 1:**
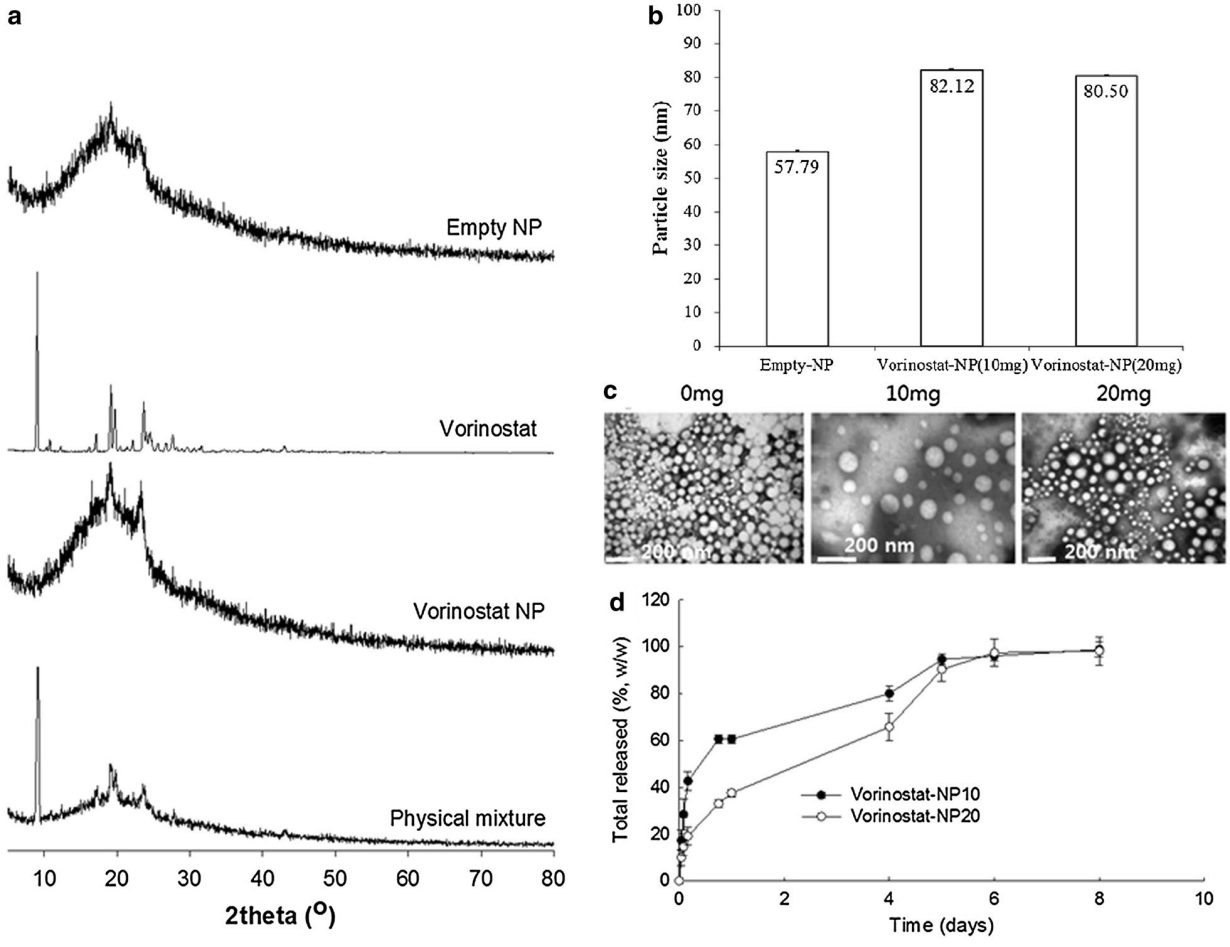
Characteristics of vorinostat-NPs. **a** XRD chromatogram; **b** average particle size; **c** TEM images; **d** drug-release kinetics. Empty-NPs were prepared similar to vorinostat-incorporated nanoparticles (vorinostat-NPs) in the absence of vorinostat

Vorinostat-incorporated nanoparticles (vorinostat-NPs) were fabricated using the nanoprecipitation method. Vorinostat and poly(dl-lactide-co-glycolide)/poly(ethylene glycol) (LGE) block copolymer was dissolved in organic solvent. Then this solution was poured into aqueous solution and the organic solvent was removed. At these procedures, there is no apparent precipitation of vorinostat in the aqueous phase. To remove remained organic solvents and emulsifier (pluronic F68), vorinostat-NPs were separated by centrifugation and washing process. As shown in Table 1, experimental drug loading of vorinostat into the nanoparticles was slightly lower than the theoretical value. This might be due to the liberation of vorinostat from the nanoparticles during the preparation procedure, thereby causing a decrease in the effective drug content. Figure 1 shows the characteristics of vorinostat-NPs. X-ray powder diffractograms (XRD) measurement of lyophilized nanoparticles was employed to confirm whether or not free drug was remained in the nanoparticle solution as shown in Fig. 1a. As shown in Fig. 1a, vorinostat alone has sharp crystalline peaks while empty nanoparticles (empty-NPs) have broad peak properties. Interestingly, vorinostat-NPs also have broad peak properties as similar to empty-NPs whereas the physical mixture of empty-NPs and vorinostat has both sharp and broad crystalline peaks. These results indicated that the intrinsic crystallinity of vorinostat was decreased by incorporation into the polymer nanoparticles and that vorinostat was molecularly distributed in the nanoparticle matrix. Furthermore, these results demonstrated that no extensive precipitation of drug has been occurred during fabrication procedure and then free drug remained was minimized. Additionally, these results also indicated that vorinostat was properly payloaded into the nanoparticles.

**Table 1:** Evaluation of drug contents of vorinostat-NPs

	Polymer/vorinostat weight ratio (mg/mg)	Drug contents (%, w/w)	
		Theoretical	Experimental
Empty	100/0	–	–
Vorinostat-NP10	100/10	9.1	8.3
Vorinostat-NP20	100/20	16.7	15.1

Vorinostat contents in the nanoparticles are listed

The average particle size was measured to investigate colloidal properties of vorinostat-NPs as shown in Fig. 1b. Particles size of nanoparticles was slightly increased when vorinostat was incorporated into the nanoparticles (Fig. 1b), but the vorinostat content of the nanoparticles did not significantly affect the change in particle diameter. As shown in Fig. 1c, empty-NPs and vorinostat-NPs have spherical shapes under transmission electron microscopy (TEM) observation with small particle sizes of <200 nm. Figure 1d shows the release properties characteristics of vorinostat from the nanoparticles. The initial burst release of the drug was observed for 1 day and, subsequently, vorinostat-NPs revealed sustained behavior over 5 days. In particular, the release rate of vorinostat was slower when the vorinostat content in the nanoparticles was higher. These phenomena might be due to that hydrophobic drugs in the nanoparticles can be aggregated at higher drug content and then aggregated drug released slowly due to the limited solubility in the aqueous phase. Gref et al. reported that increased drug contents of hydrophobic drugs into core–shell type nanospheres lead to aggregation or crystallization in the cores of the nanospheres [28]. The crystallization or aggregation of hydrophobic drug in the core of the nanospheres led slow rate of dissolution and diffusion of drugs into the aqueous phase [28]. Then, the release rate of hydrophobic should be slower at higher drug loading than lower drug loading.

### In vitro anticancer activity of vorinostat-incorporated nanoparticles

**Fig. 2:**
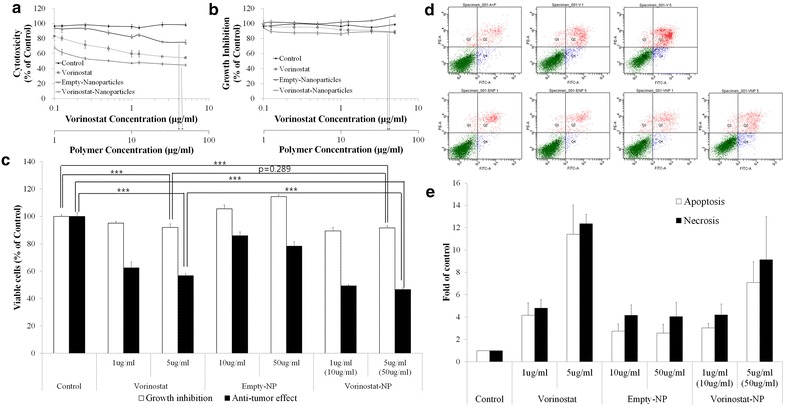
Anticancer activity of vorinostat and vorinostat-NP against HuCC-T1 cells in vitro. 3 × 10^5^ cells for cytotoxicity (**a**) and 3 × 10^4^ cells for growth inhibition (**b**) test were exposed to vorinostat or vorinostat-NPs for 24 h, respectively. For cytotoxicity study, serum-free media were used and grow inhibition test was performed with 10 %-FBS supplemented media. **c** Comparison of cytotoxicity and growth inhibition of HuCC-T1 cells following treatment with vorinostat or vorinostat-NPs at 1and 5 μg/ml. **d**, **e** Apoptosis and necrosis analysis of HuCC-T1 cells. For apoptosis or necrosis, 1 × 10^6^ cells were exposed to vorinostat or vorinostat-NPs for 24 h. FITC-conjugated Annexin V was used to analyze apoptosis and PI was used to analyze necrosis

Figure 2 shows the anticancer activity of vorinostat and vorinostat-NPs against HuCC-T1 cells. For cytotoxicity index, viability of cells was checked in the serum-free media as shown in Fig. 2a. The viability of the cells was slightly decreased according to the increase of vorinostat concentration. In particular, vorinostat-NPs has higher cytotoxicity than vorinostat alone even though empty nanoparticles have small effect on the viability of cells. In growth inhibition study, both vorinostat and vorinostat-incorporated nanoparticles have low inhibitory effects on the growth of HuCC-T1 cells as shown in Fig. 2b. These results were compared at vorinostat concentration of 1 and 5 μg/ml as shown in Fig. 2c. Both vorinostat and vorinostat nanoparticles has small inhibitory effects on the cell growth at 1 and 5 μg/ml while empty nanoparticles did not affect to the growth of HuCC-T1 cells. Figure 2d shows the apoptosis/necrosis analysis of HuCC-T1 cells upon treatment with vorinostat and vorinostat-incorporated nanoparticles. As shown in Fig. 2d, e, the apoptosis and necrosis of HuCC-T1 cells were significantly increased at 5 μg/ml of a vorinostat concentration when vorinostat or vorinostat-incorporated nanoparticles were treated. The empty nanoparticles showed minimal effect to cells.

**Fig. 3:**
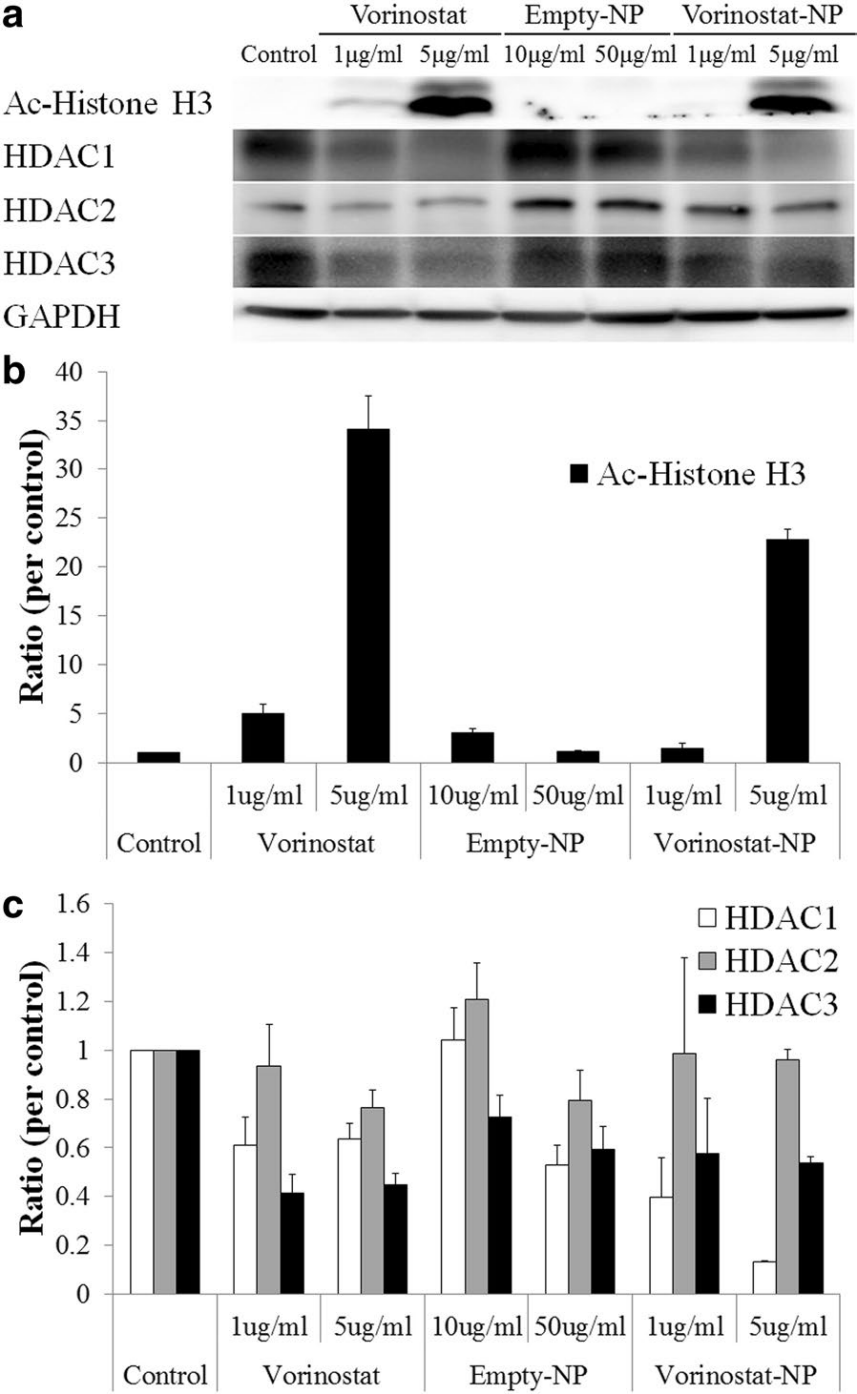
Western blot analysis of Ac-histone H3 and HDAC expression after treatment with vorinostat or vorinostat-NPs. **a** expression of Ac-histone H3 and HDACs; **b** quantitation of Ac-histone H3 expression; **c** quantitation of HDAC expression

Figure 3 shows the western blot analysis of molecular signals in HuCC-T1 cells upon treatment with vorinostat and vorinostat-NPs. As shown in Fig. 3a, acetylated histone H3 was evidently increased upon treatment with 5 μg/ml vorinostat and vorinostat-NPs, whereas empty-NPs have no effect on this signal. As shown in Fig. 3b, quantitative analysis showed a significant increase in ac-histone H3 expression in HuCC-T1 cells, indicating that vorinostat-incorporated nanoparticles were effective in acetylation of Histone H3 as well as vorinostat itself. Furthermore, treatment of vorinostat-incorporated nanoparticles were also effective in decreasing expression of HDAC 1 and HDAC 3 as well as vorinostat itself even though HDAC2 expression was almost similar or slightly higher than control. In fact, HDAC2 and 3 expression of vorinostat-incorporated nanoparticles were slightly higher than vorinostat itself at 5 μg/ml concentration (Fig. 3a, c). These results might be due to the sustained release properties of nanoparticles, i.e. nanoparticles slowly released the drug into the cell culture medium and lower concentration of intact vorinostat can affected to the cellular expression of HDAC2 and 3. Interestingly, empty-NPs affected HDAC expression in cancer cells at high concentrations (50 μg/ml). Because the reason for these results is not clear, further investigations are required in the future. One possible explanation of these results is that high concentrations of empty-NPs show some cytotoxic effects in the in vitro cell culture environment, even though LGE block copolymer has already been approved as a biocompatible polymer and approved for human use by the US FDA [29].

**Fig. 4:**
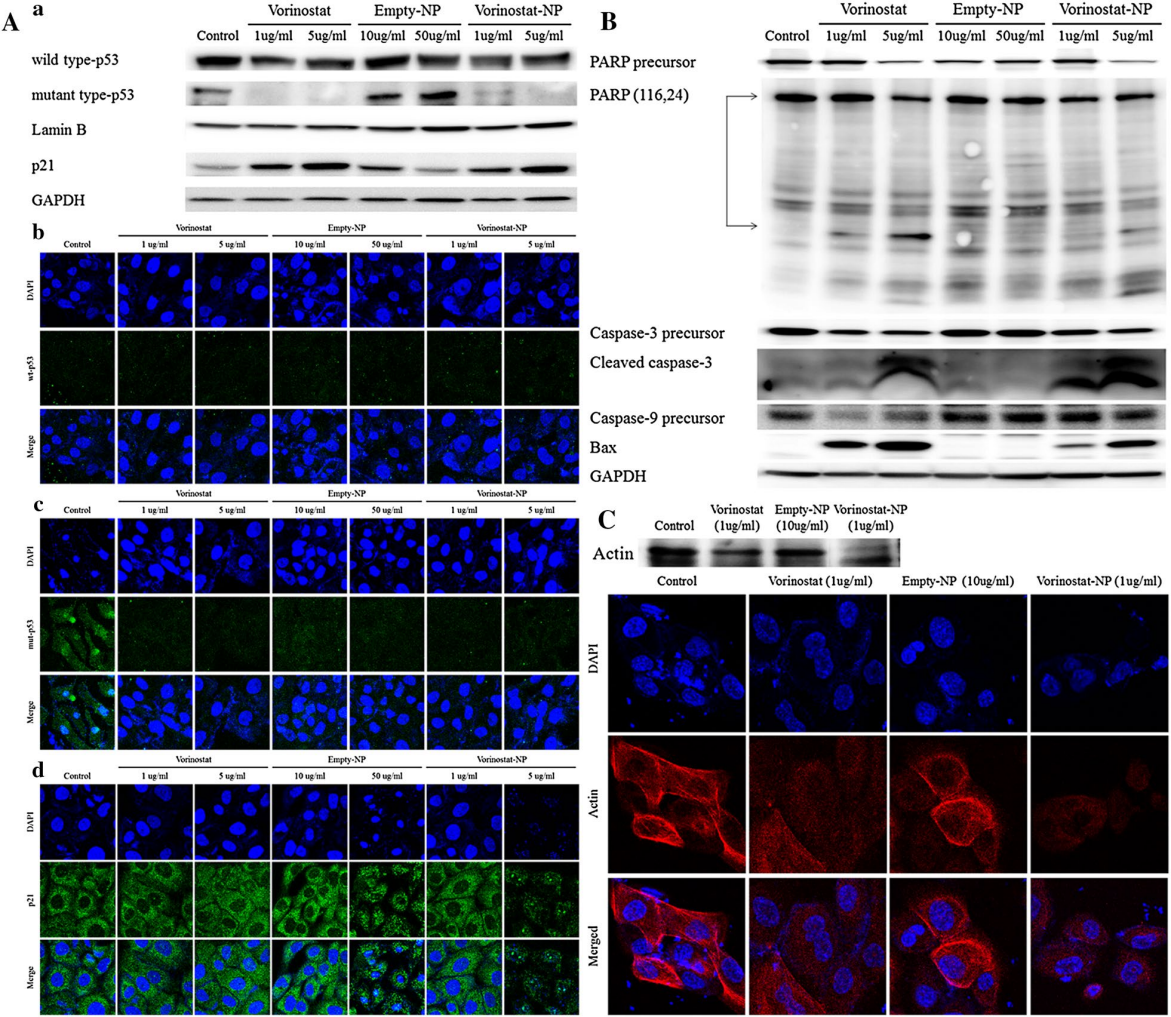
Expression of signals after treatment with vorinostat and vorinostat-NPs. **A** *a* Western blot analysis of apoptotic signal expression in HuCC-T1 cells following treatment with vorinostat or vorinostat-NPs.* b*–*d* Immunocytochemistry (×1200) of wild-type p53, mutant p53 and p21 of HuCC-T1 cells after treatment with vorinostat or vorinostat-NPs. **B** Western blot analysis of PARP precursor, PARP, caspase-3 precursor, cleaved caspase-3, caspase-9 precursor and Bax. **C** Western blot analysis and immunocytochemistry of actin

Figure 4A shows that the level of mutant p53 was significantly decreased both by vorinostat and vorinostat-NPs, whereas that of wild-type p53 was not significantly changed. The expression of p21 was significantly increased upon treatment with both vorinostat and vorinostat-NPs. Upon immunocytochemical staining of HuCC-T1 cells, a decrease in mutant-type p53 and an increase in p21 were also observed. Expression of each protein was normalized to the GAPDH as cytosolic control and lamin B as nuclear control. Figure 4B shows apoptosis signals in HuCC-T1 cells. As shown in Fig. 4B, a decrease in the level of poly-ADP ribose polymerase (PARP) precursor and an increase in cleaved PARP (at 24 kDa) were observed. Furthermore, decreases in caspase-3 and -9 precursors were also observed upon treatment with vorinostat and vorinostat-NPs. And, the level of cleaved caspase-3 was increased, as shown in Fig. 4B. Including the result of Bax expression, these results indicated that vorinostat-NPs induced apoptosis and affected the expression of apoptotic molecular signals to the same extent as did vorinostat. On treatment with vorinostat or vorinostat-NPs, actin was disrupted in HuCC-T1 cells as shown in Fig. 4C, indicating that vorinostat-NPs have a similar effect on apoptosis and protein changes at the cellular level as does vorinostat.

### In vivo antitumor activity of vorinostat-incorporated nanoparticles

**Fig. 5:**
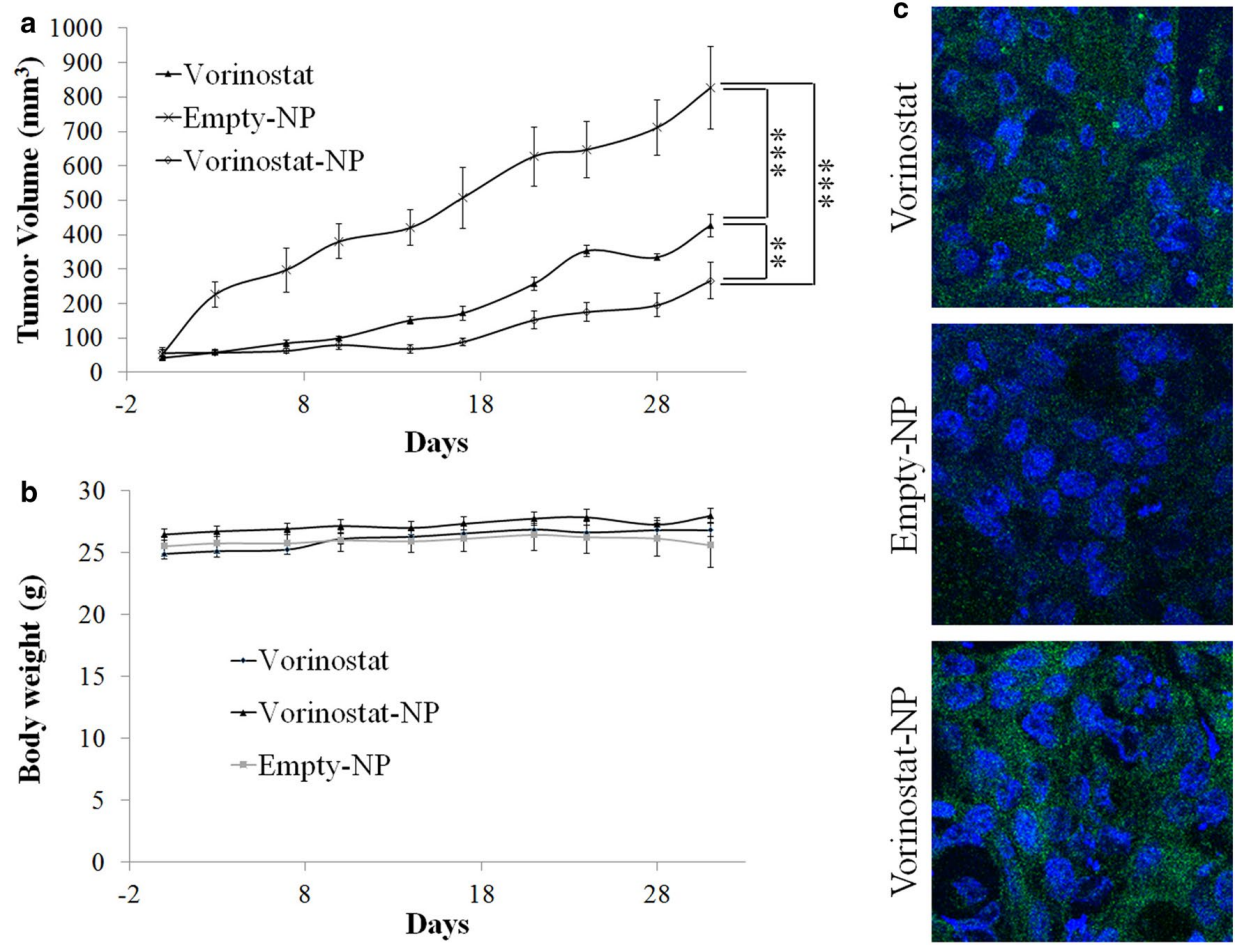
Antitumor activity of vorinostat or vorinostat-NP in HuCC-T1 tumor xenograft mice model. (dose 50 mg vorinostat/kg) **a** tumor volume;** b** body weight;** c** TUNEL assay (×400) of extracted tumor tissues. HuCC-T1 human CCA cells (1 × 10^7^) were implanted into the back of BALb/C nude mouse. 2 weeks later, vorinostat, empty-NPs or vorinostat-NPs were injected subcutaneously beside the solid tumor and the day of drug injection was set as day 0. Tumor volume was calculated using the formula *V* = (*a* × [*b*]^2^)/2, with *a* being the largest and *b* being the smallest diameter. For TUNNEL assay, tumors were isolated and fixed with 4 % formamide after 30 days of injection

**Fig. 6:**
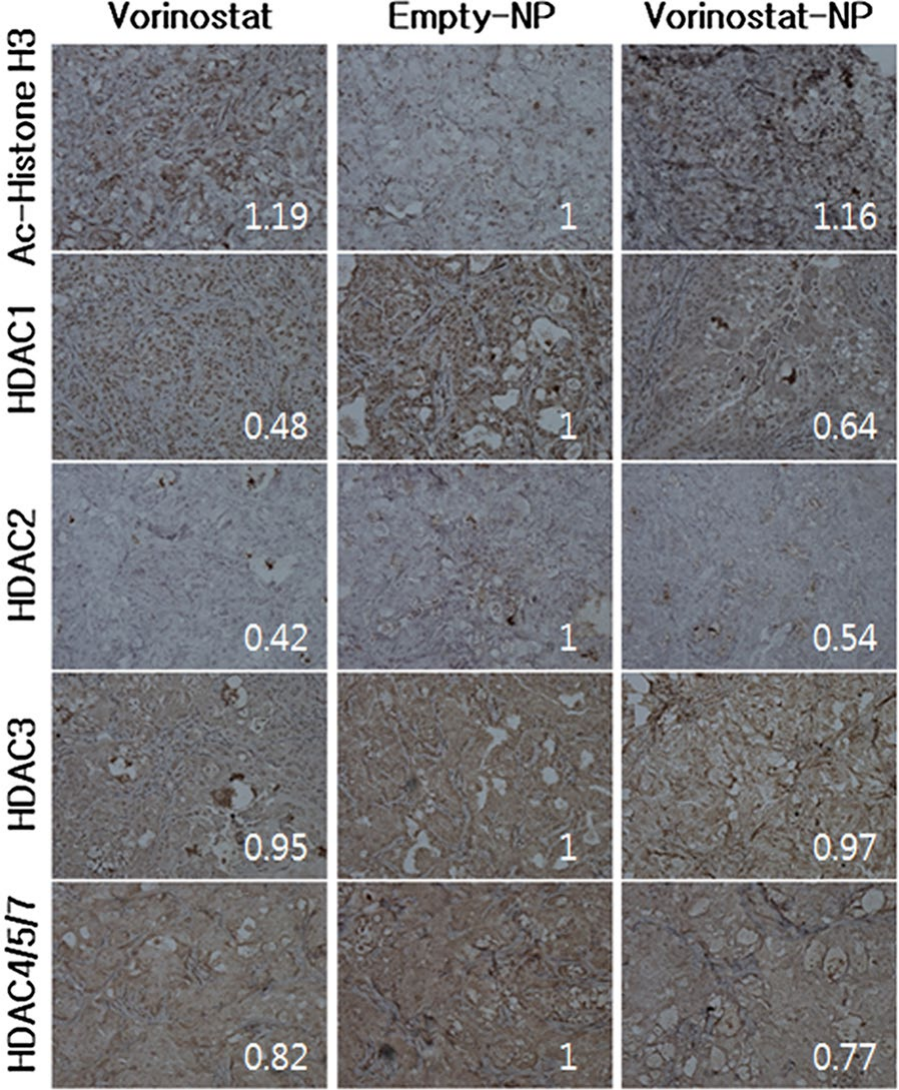
Immunohistochemistry (×400) of tumor tissues from HuCC-T1 cell bearing xenograft mouse model. To study HDAC expression, tumor tissues were stained with acetyl histone H3, HDAC1, HDAC2, HDAC3, and HDAC4/5/7 antibodies

The increase in tumor volume and changes in body weight were monitored. As shown in Fig. 5a, the size of the tumor rapidly increased with time following treatment of empty-NPs. However, the volume of the tumor was significantly suppressed by treatment of vorinostat or vorinostat-NPs. Body weight did not significantly change with any of the treatments, indicating that neither vorinostat nor nanoparticles were significantly toxic to the mice, as shown in Fig. 5b. Interestingly, vorinostat-NPs showed higher efficacy of tumor growth inhibition: tumor volume following treatment of vorinostat-NPs was almost 50 % smaller than following treatment of vorinostat. Terminal deoxynucleotidyl transferase dUDT nick-end-labeling (TUNEL) staining of solid tumors supported these results, as shown in Fig. 5c: upon treatment of vorinostat-NPs, apoptosis was higher than treatment of vorinostat, whereas minimal apoptosis was seen upon treatment of empty-NPs. Interestingly, the expression of ac-histone H3 in tumor tissue was significantly increased upon treatment with vorinostat-NPs compared to empty-NPs and vorinostat as shown in Fig. 6. Furthermore, the expression levels of HDAC 1, 2, 3 and 4/5/7 were relatively decreased upon treatment of vorinostat and vorinostat-NPs, compared to treatment of empty-NPs. These results indicated that subcutaneous injection of vorinostat-NPs has similar or higher antitumor activity compared to vorinostat.

**Fig. 7:**
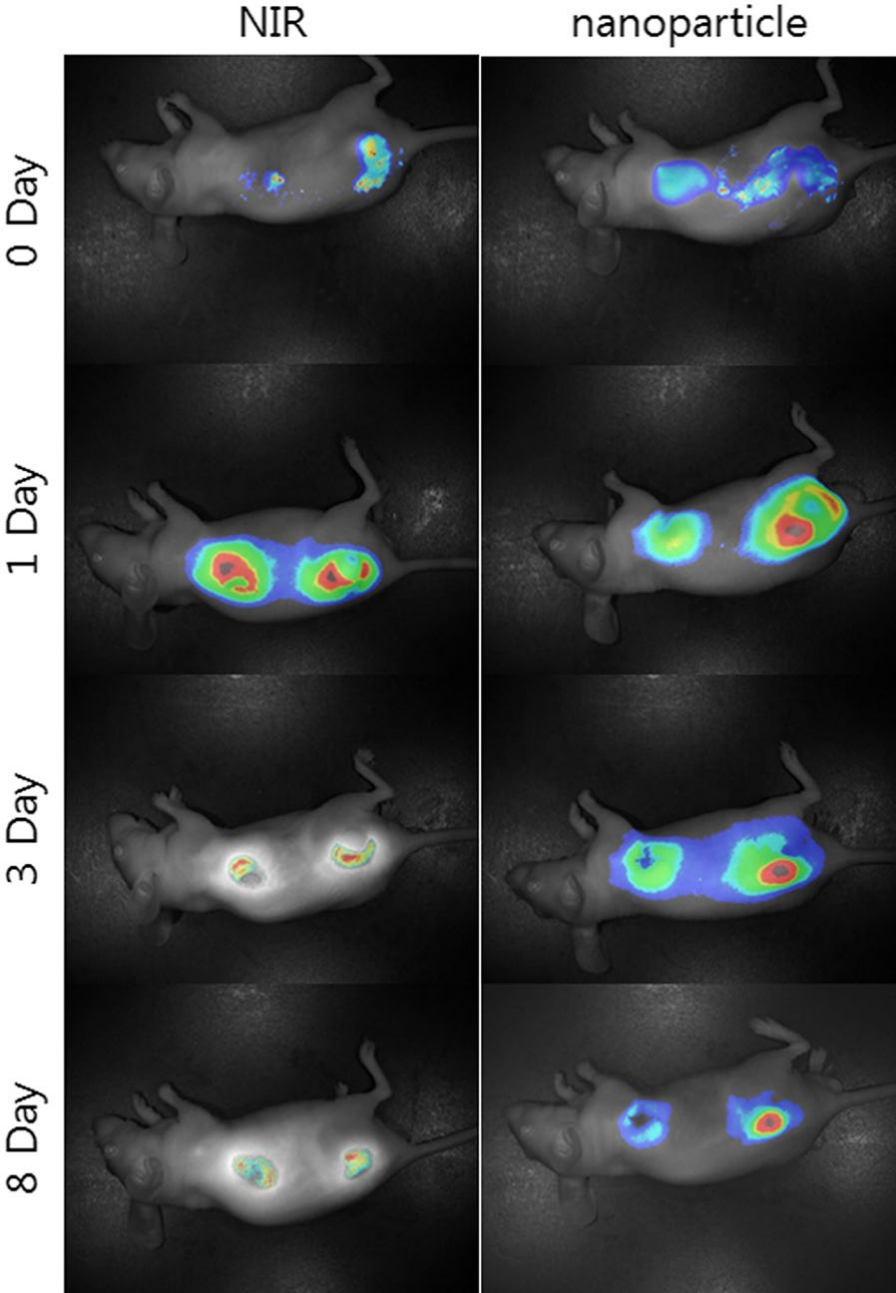
In vivo fluorescence imaging of HuCC-T1 tumor xenograft mice model. NIR dye-incorporated NPs were simultaneously injected into a normal region and beside the tumor region of the back of mouse. Mouse were observed with the Maestro 2™ In Vivo imaging system at 780 nm

To clarify the reason for the higher antitumor activity of the nanoparticles, near infrared (NIR)-dye-conjugated nanoparticles (NIR-NPs) were subcutaneously injected into a normal region as well as the site of the tumor, as shown in Fig. 7. Intact NIR-dye treated mice showed a rapid decrease in both the normal region and the tumor site. However, nanoparticle treated mice showed quite different results, i.e. nanoparticles remained longer at the tumor site than at the normal region. In particular, the strongest fluorescence intensity was observed at the center of the solid tumor at 1 day after treatment with NIR-NPs, whereas intact NIR-dye revealed the strongest fluorescence intensity in the region surrounding the solid tumor. The treatment of NIR-NPs revealed strong fluorescence intensity after 8 days for injection. These results confirmed that vorinostat-NPs have higher antitumor activity in vivo than does vorinostat.

## Discussion

HDAC expression in cancer cells has a critical role in remodeling of chromatin structure, gene expression, cell cycle regulation and differentiation [30, 31]. Increased HDAC activity is known to result in malignant tumor behavior [30]. In particular, Morine et al. reported that HDAC1 expression in intrahepatic CCA is significantly correlated with the stage of carcinogenesis and is related to malignant behaviors of cancer, such as angiogenesis, lymph node metastasis and vascular invasion [30]. They found that the survival rate of the HDAC1-positive group was significantly worse than that of the negative group. Furthermore, HDAC6 is also known to have a strong relationship with the stage of CCA and can thus be considered a clinic-pathological parameter for CCA [32]. Higher HDAC expression is associated with shorter survival times in gastric cancer patients and is regarded as an independent prognostic marker for gastric cancer [33]. Inhibition of the molecular action of HDAC using HDAC inhibitors is a promising candidate for cancer chemotherapy [34, 35].

HDAC inhibitors exert anticancer activities on human cancer cells through cell cycle arrest, growth arrest, activation of apoptotic pathways, autophagic cell death, reactive oxygen species (ROS)-mediated cell death and mitotic cell death etc. [35, 36]. HDAC inhibitors tightly bind to DNA histones and prevent the transcription/expression of tumor suppressor genes by inducing histone acetylation [35, 36]. Several HDAC inhibitors which inhibits class I and II HDACs [36] show minimal intrinsic toxicity to human bodies but show dramatic anticancer efficacy for cancer [37]. In clinical trials, oral administration of vorinostat with promising anticancer activity was well tolerated in patients with GI cancers [3]. In animal tumor xenograft studies, intraperitoneal injection of vorinostat induced tumor necrosis and inhibited the growth of colon tumors through the inhibition of different subtypes of HDACs [5].

In our study, we fabricated vorinostat-NPs for treatment of CCA. As shown in Fig. 1, vorinostat-NPs have spherical shapes and small diameter <100 nm. They showed sustained drug release behavior over 5 days as shown in Fig. 1d. Especially, nanoparticles having higher vorinostat contents (vorinostat-NP20) showed slower release kinetic. These results might be due to that hydrophobic drug can be aggregated at higher drug loading contents and release of aggregated drug can be delayed compared to the nanoparticles with lower drug contents (vorinostat-NP10). These phenomena were frequently reported by several investigators. To assess biological activity, vorinostat-NPs were treated to HuCC-T1 cells and their anticancer activity was compared to vorinostat itself at in vitro and in vivo. Vorinostat-NPs as well as vorinostat properly inhibited the growth of HuCC-T1 cells in vitro and the growth of tumor volume in vivo through inhibition of HDAC expression in the HuCC-T1 cells and tumor tissues, as shown in Figs. 3, 5 and 6. Furthermore, vorinostat-NPs have higher antitumor activity compared to vorinostat, due to the sustained release properties of nanoparticles. The apoptotic signals of HuCC-T1 cells were also significantly altered upon treatment with vorinostat or vorinostat-NPs in vitro and in vivo as shown in Figs. 3, 4 and 5. As shown in Fig. 4, levels of mutant p53 were significantly decreased with little change in wild-type p53 and this suppression is correlated with the expression of PARP/cleaved caspase-3. Other researchers have reported that the expression of mutant p53 was significantly decreased in a dose- and time-dependent manner [38, 39]. For example, Yan et al. found that disruption of HDAC8 expression significantly inhibits proliferation of cancer cells having mutant-type p53 irrespective of wild-type p53. In their results, colony formation of mutant-type p53 cell lines SW480 was remarkably decreased by treatment of vorinostat while wild-type p53 cell lines HCT116 showed little changes [40]. Furthermore, both vorinostat-NPs and vorinostat were able to arrest cell growth and induced apoptosis as shown in Figs. 3 and 4. And, they increased the expression of p21, a cyclin-dependent kinase inhibitor I, in a dose-dependent manner and this is correlated with other apoptosis signals. Thenaa et al. also reported that vorinostat inhibits mammary cell growth through altered p21 expression and cell cycle arrest [41]. Our results showed that vorinostat-incorporated nanoparticles as well as vorinostat itself also affects in induction of apoptotic signals, suppression of mutant-type p53, up-regulation of p21 and disruption of actin in HuCC-T1 cells. Furthermore, vorinostat-incorporated nanoparticles were also higher efficacy than vorinostat itself at in vivo animal tumor xenograft study. Treatment with vorinostat or vorinostat-incorporated nanoparticles increased acetylation of histone H3 (Ac-Histone H3) and then decreased HDAC expression as shown in Fig. 6. Di Gennaro et al. also reported that subcutaneous injection of vorinostat at a dose of 100 mg/kg against SW620 colorectal cancer xenografts increases Ac-Histone H3 and showed synergized effect with capecitabine in the inhibition of tumor growth [42]. Furthermore, intravenous injection of vorinostat or vorinostat-incorporated nanoparticles is known to sensitize radiotherapy and then effectively inhibit growth of PC3 tumor xenograft in mice [43].

Hydrogels or nanocarriers are known to improve antitumor activity of vorinostat in vivo animal tumor xenograft model [44–46]. Vorinostat-NPs caused antitumor activity, apoptotic expression in TUNEL assay and inhibitory activity of HDAC expression compared with vorinostat itself, as shown in Figs. 5 and 6. Antitumor activity of drugs can be improved at in vivo circumstances by use of biodegradable polymers for controlled drug release [41]. Therefore, these results can be explained by the sustained release properties of vorinostat-NPs. Li et al. reported that biodegradable thermosensitive hydrogel enhances the therapeutic efficacy of vorinostat and significantly inhibited intratumoral angiogenesis [44]. Furthermore, Mohamed et al. reported that polymeric micelles significantly enhance half-lives in blood and bioavailability of vorinostat in rats by intravenous injection and oral administration [45]. Nanocarriers also increase the half-life of vorinostat in blood and improve the antitumor activity of vorinostat [46]. Gref et al. reported potential of long blood circulation of core–shell type nanospheres composed of PLGA-PEG block copolymer rather than plain nanoparticles [28]. Systemic approach of chemotherapeutic agents is known to have limited clinical benefit due to the difficulties of drug delivery to CCA tumor [20, 21, 47]. For this reason, alternative treatment regimen is required to deliver the anticancer drugs to CCA tumor. Therefore, we focused on the possibility of drug delivery to CCA tumor by local administration of vorinostat-incorporated nanoparticles. Practically, growth of tumors originated in HuCC-T1 cells in the back of the mice was effectively suppressed compared to vorinostat itself and empty nanoparticle treatment as shown in Fig. 5. To clarify vorinostat delivery to tumor tissues, NIR-dye was physically incorporated into the nanoparticles as similar to vorinostat and injected beside tumor tissues. As shown in Fig. 7, it is likely that nanoparticles were efficiently delivered to tumor tissues compared to free NIR-dye. Furthermore, NIR-NPs stayed longer in the tumor tissue than free NIR-dye. Practically, NIR-NPs were rapidly cleared from normal region but not in tumor region while free NIR-dye was rapidly cleared both in normal and tumor region. The reason of improved antitumor activity of vorinostat nanoparticles compared to vorinostat itself can be explained by these results. Other researcher also reported that nanoparticles can be stayed longer in the injection site and efficiently delivered to tumor tissues compared to free NIR-dye [48]. In other words, enhanced permeation and retention effect of macromolecules and nanomedicines in the tumor tissues also can be considered to explain these results [49, 50]. In our results, vorinostat-NPs showed higher anticancer activity than intact vorinostat and have higher efficacy in the drug delivery to tumor tissue. We suggest that vorinostat nanoparticles are promising candidate to treat CCA.

## Conclusion

We prepared vorinostat-NPs using biodegradable block copolymer for anticancer therapy in HuCC-T1 CCA cells. Vorinostat-NPs have similar anticancer activities in terms of growth inhibition, apoptosis and inhibition of HDAC expression in vitro to that of vorinostat alone. However, vorinostat-NPs show improved antitumor activity in xenograft mice model and a higher inhibition rate of HDAC expression in vivo. The higher anticancer activity of vorinostat-NPs can be explained by NIR-NPs, i.e. NIR-NPs were remained in the tumor tissue longer than did free NIR dye. We suggest that vorinostat nanoparticles can be used as a promising vehicle for HDAC-targeted chemotherapy in CCA cells.

## Methods

### Materials

Vorinostat was purchased from LC Labs. Co. (Woburn, MA, USA). LGE copolymer (Resomer^®^ RGP d 50105) was purchased from Boehringer Ingelheim Pharma GmbH & Co. (Ingelheim am Rhein, Germany). Pluronic F68, dimethyl sulfoxide (DMSO) and acetone were purchased from Sigma-Aldrich Chem. Co. (St. Louis, MO, USA). Dialysis membranes with molecular weight cutoffs of 8000 g/mol were purchased from Spectra/PorTM (Spectrum Laboratories Inc, Rancho Dominguez, CA, USA). RPMI1640 media, fetal bovine serum (FBS) and all cell culture components were purchased from Life Technologies (Grand Island, NY, USA). All reagents and organic solvents used were of extra-pure grade.

### Fabrication of vorinostat-incorporated nanoparticles

One hundred milligrams of LGE were dissolved in 10 ml acetone. Ten and twenty mg of vorinostat was dissolved in 0.2 and 0.4 ml of DMSO, respectively. Then, vorinostat solution was mixed with LGE/acetone solution. The mixed solution was dropped in 20 ml of deionized water [Pluronic F68, 0.1 % (w/v)] for 10 min and then the organic solvent was evaporated under vacuum. The nanoparticle solution was recovered by ultra-centrifugation at 100,000×*g* (Supra 30 K, Vacuum High Speed Centrifuge, Hanil Science Industrial Co. Ltd., Incheon, Korea). Subsequently, harvested nanoparticles were washed with 10 ml of deionized water and then harvested again by ultra-centrifugation. The washing procedure was repeated three times. The resulting nanoparticles were reconstituted in deionized water or lyophilized. To measure vorinostat content in the nanoparticles, 5 mg of lyophilized nanoparticles were dissolved in DMSO. The drug content and loading efficiency of vorinostat in the vorinostat-NPs was evaluated using the Flexar high-performance liquid chromatography (HPLC) system (Perkin-Elmer Life and Analytical Sciences, Waltham, MA, USA).

Drug concentrations were determined using the HPLC system as follows: the Flexar HPLC system was equipped with a Solvent Manager 5-CH degasser, an autosampler, a quaternary LC pump, a column oven and an UV/VIS detector. Chromatography was performed on a guard column (SecurityGuard^®^ Guard Cartridge Kit; Phenomenex, Torrance, CA, USA) and a C18 column (Brownlee C18^®^, 5 micrometer, 150 × 4.6; Perkin Elmer) at 37 °C. Vorinostat was eluted isocratically with mobile phase (acetonitrile/0.1 % formic acid at a ratio of 22/78) at a flow rate of 1 ml/min and monitored at 241 nm. Chromatograms were recorded and integrated with the Chromera 2.1 system software (Perkin Elmer Life and Analytical Sciences, Waltham, MA, USA).

$$ \begin{aligned} {\text{Drug content }} & = {\text{ [(Drug weight in the nanoparticles)}}/({\text{weight of nanoparticles}}) ]\times 100 \\ {\text{Loading efficiency }} & = {\text{ [(Residual drug in the nanoparticle)}}/({\text{initial feeding amount of drug}}) ]\times 100 \\ \end{aligned} $$

### Characterization of vorinostat-incorporated nanoparticles

The morphology of the nanoparticles was observed using TEM (JEM-2000 FX II microscope, JEOL, Tokyo, Japan). The nanoparticle solution was dropped onto a carbon film coated on a copper grid and then the nanoparticles were negatively stained with phosphotungstic acid (0.05 % w/w). TEM observation was performed at an accelerating voltage of 80 kV. Particle size was measured using the Nano-ZS apparatus (Malvern Instruments, Malvern, UK). Nanoparticles were reconstituted in deionized water (nanoparticle concentration 0.1 mg/ml) and then used to determine particle size. The crystallinity of vorinostat and vorinostat-NPs were analyzed using XRD (Rigaku D/Max-1200, Rigaku, Tokyo, Japan) equipped with Ni-filtered Cu Ka radiation (40 kV, 20 mA). The vorinostat powder and lyophilized nanoparticle solid were used to measure crystallinity using XRD.

### Drug release study

Drug release testing was performed using phosphate-buffered saline (PBS; 10 mM, pH 7.4) solution at 37 °C. Five milligrams of nanoparticles in 1 ml of deionized water were added to 4 ml of PBS and this solution was then introduced into a dialysis tube. This dialysis tube was immersed in a 100 ml bottle with 95 ml of PBS. Whole media were taken at predetermined time intervals and exchanged with fresh PBS. The concentration of the released drug was measured using the HPLC system. The percentage of released drug was calculated from following equation: [(amount of released drug/total weight of drug in the nanoparticles) × 100].

### Cell culture

HuCC-T1 cell line was obtained from the Health Science Research Resources Bank (Osaka, Japan) and maintained with RPMI1640 medium supplemented with 10 % heat-inactivated FBS and 1 % penicillin/streptomycin at 37 °C in a humidified atmosphere containing 5 % CO_2_.

### Cell cytotoxicity and growth inhibition study

HuCC-T1 cells were seeded in 24-well plates at a density of 3 × 10^4^ and 3 × 10^5^ cells per well for the growth inhibition and cytotoxicity assays, respectively. Following this, each plate was incubated overnight in a CO_2_ incubator. Vorinostat in DMSO and vorinostat-NPs were diluted with RPMI1640 medium containing 10 % FBS for the growth inhibition assay at various concentrations and then added to HuCC-T1 cells in 24-well plates following 24 h incubation. The cytotoxicity assay was carried out using serum-free RPMI1640 media. The control was treated with 0.1 % (v/v) DMSO. Cells were trypsinized, harvested and resuspended in PBS. Trypan blue was added and the number of cells was counted using the Countess™ Automated Cell Counter (Invitrogen, Carlsbad, CA, USA). The reduction of viable cells by treatment of vorinostat or vorinostat-incorporated nanoparticles compared to control treatment was calculated and expressed as mean ± SD.

### Apoptosis and necrosis analysis

HuCC-T1 cells were seeded in 6-well plates at a density of 1 × 10^6^ cells per well and exposed to various concentrations of vorinostat and vorinostat-NPs for 24 h. The cells were harvested, washed with PBS, resuspended in 500 μl binding buffer and stained with FITC-conjugated Annexin V for apoptosis analysis and with PI for necrosis analysis. These cells were analyzed by flow cytometry (BD biosciences, San Jose, CA, USA).

### Western blot analysis and immunocytochemistry

HuCC-T1 cells were seeded in 6-well plates at a density of 1 × 10^6^ cells per well and exposed to various concentrations of vorinostat and vorinostat-NPs for 24 h. Cells were trypsinized and washed with cold PBS. The cells were collected by centrifugation and lysed in lysis buffer containing protease inhibitors [50 mM Tris, 150 mM NaCl, 1 % NP-40, 0.5 % deoxycholic acid, 0.1 % sodium dodecyl sulfate (SDS)] with phenylmethylsulfonyl fluoride and a protease inhibitor cocktail (Roche Diagnostics, Basel, Switzerland). The cell suspension was cleared by centrifugation at 14,000×*g* for 30 min at 4 °C and then supernatant or cell lysates were collected. The protein concentration was determined using the BCA Protein Assay kit (Pierce, Rockford, IL, USA).

For western blotting, 50 μg protein was subjected to SDS-polyacrylamide gel electrophoresis (SDS-PAGE), transferred to a polyvinyl difluoride membrane, blocked with 5 % skim milk in TBS-T and probed with an appropriate primary antibody followed by a secondary HRP-conjugated antibody. Proteins were detected by chemiluminescence. Proteins were quantified by digital analyses.

### Antitumor activity of vorinostat-incorporated nanoparticles against the animal tumor xenograft model

To assess the antitumor activity of vorinostat-NPs, a tumor xenograft model was prepared by subcutaneous injection of HuCC-T1 cells into the backs of nude mice. HuCC-T1 cells (1 × 10^7^ cells) in a total volume of 100 μl were subcutaneously injected into the backs of male nude mice (5-week-old and 20–25 g in weight; Orient, Seongnam, South Korea). When the solid tumor reached approximately 4–5 mm in diameter, empty-NPs, vorinostat and vorinostat-NPs were injected subcutaneously adjacent to the solid tumor. Treatment dose was adjusted to 1 mg vorinostat (50 mg/kg). A total of 18 mice were divided into three groups, as follows: (1) vorinostat-injected, (2) empty-NPs injected and (3) vorinostat-NP injected. Body weight and tumor volume were measured twice a week, starting on the first day of treatment. Two perpendicular diameters of the tumor were measured and tumor volume was calculated using the formula *V* = (*a* × [*b*]^2^)/2, with *a* being the largest and *b* being the smallest diameter. The animal study was carried out according to the guidelines of the Animal Treatment and Research Council of Pusan National University.

### Immunohistochemistry

After 30 days of injection, tumors were isolated and fixed in 4 % formamide, paraffin-embedded and sliced for hematoxylin and eosin (H&E) staining or for the TUNEL assay. For immunohistochemical staining of the tumors, acetyl histone H3 antibody was diluted to 1:500 and HDAC1 antibody was dilued to 1:100. HDAC2, HDAC3, and HDAC4/5/7 antibodies were diluted to 1:200. Staining was performed using an Envision kit (Life Technologies, Carlsbad, CA, USA) according to the manufacturer’s protocol.

### Fluorescence imaging of solid tumor-bearing mice

To study biodistribution of vorinostat-NPs, hydrophobic NIR-dye (XenoLight DiR, Caliper Lifesciences, MA 01748-1668, USA) was incorporated into the nanoparticles. Four milligrams of hydrophobic NIR-dye was dissolved in 0.2 ml of DMSO and mixed with 100 mg of LGE dissolved in acetone. This solution was dropped in 20 ml of deionized water [Pluronic F68, 0.1 % (w/v)] for 10 min and then organic solvent was evaporated under vacuum. After that, hydrophobic NIR-NPs were harvested by same procedure as described above. To measure content of hydrophobic NIR-dye in the nanoparticles, hydrophobic NIR-NPs were dissolved in 10 ml of DMSO and the concentration was measured with fluorescence spectrophotometer (RF-5301 fluorescence spectrofluorophotometer, Shimadzu, Tokyo, Japan). The content of hydrophobic near-infrared was calculated to 3.6 % (w/w).

For tumor imaging, NIR-NPs (50 mg/kg) were injected subcutaneously beside tumor tissue. To compare uptake of nanoparticles at tumor tissue and normal tissue, same quantity of NIR-NPs was also injected subcutaneously in the normal region. Mouse was observed using the Maestro 2™ In Vivo imaging system (Cambridge Research and Instruments, Inc., Woburn, MA 01801, USA) at 780 nm.

### Quantification of image intensity

Quantification of staining intensities was calculated using the ImageJ (ver 1.42q) software (NIH, Bethesda, MD, USA).

### Statistical analysis

Statistical analyses of the data from treated and untreated cells were performed using the Student’s t test. A p value <0.05 was considered to be statistically significant.

## Abbreviations

vorinostat-NPs: vorinostat-incorporated nanoparticles
HDAC: histone deacetylase
HDACi: histone deacetylase inhibitor
GI: gastrointestinal
CCA: cholangiocarcinoma
LGE: poly(dl-lactide-co-glycolide)-b-poly(ethylene glycol)
DMSO: dimethyl sulfoxide
FBS: fetal bovine serum
TEM: transmission electron microscopy
XRD: X-ray powder diffractograms
PARP: poly-ADP ribose polymerase
SDS: sodium dodecyl sulfate
PAGE: polyacrylamide gel electrophoresis
TUNEL: terminal deoxynucleotidyl transferase dUDP nick-end-labeling
NIR: near-infrared
NIR-NPs: NIR-dye conjugated nanoparticles
empty-NPs: empty nanoparticles
